# *EGFR* gene amplification is relatively common and associates with outcome in intestinal adenocarcinoma of the stomach, gastro-oesophageal junction and distal oesophagus

**DOI:** 10.1186/s12885-016-2456-1

**Published:** 2016-07-07

**Authors:** Eva-Maria Birkman, Annika Ålgars, Minnamaija Lintunen, Raija Ristamäki, Jari Sundström, Olli Carpén

**Affiliations:** Department of Pathology, University of Turku and Turku University Hospital, TYKS-SAPA, Turku, Finland; Department of Oncology, University of Turku and Turku University Hospital, Turku, Finland; MediCity Research Laboratory, University of Turku, Turku, Finland; Auria Biobank, Turku, Finland

**Keywords:** EGFR, HER2, Silver *in situ* hybridization, Gene amplification, Gastric cancer

## Abstract

**Background:**

Approximately 50 % of gastric adenocarcinomas belong to a molecular subgroup characterised by chromosomal instability and a strong association with the intestinal histological subtype. This subgroup typically contains alterations in the receptor tyrosine kinase–RAS pathway, for example *EGFR* or *HER2* gene amplifications leading to protein overexpression. In clinical practice, HER2 overexpressing metastatic gastric cancer is known to respond to treatment with anti-HER2 antibodies. By contrast, anti-EGFR antibodies have not been able to provide survival benefit in clinical trials, which, however, have not included patient selection based on the histological subtype or *EGFR* gene copy number analysis of the tumours. To examine the role of EGFR as a potential biomarker, we studied the prevalence, clinicopathological associations as well as prognostic role of EGFR and HER2 expression and gene amplification in intestinal adenocarcinomas of the stomach, gastro-oesophageal junction and distal oesophagus.

**Methods:**

Tissue samples from 220 patients were analysed with EGFR and HER2 immunohistochemistry. Those samples with moderate/strong staining intensity were further analysed with silver *in situ* hybridization to quantify gene copy numbers. The results were associated with clinical patient characteristics and survival.

**Results:**

Moderate/strong EGFR protein expression was found in 72/220 (32.7 %) and *EGFR* gene amplification in 31/220 (14.1 %) of the tumours, while moderate/strong HER2 protein expression was detected in 31/220 (14.1 %) and *HER2* gene amplification in 29/220 (13.2 %) of the tumours. *EGFR* and *HER2* genes were co-amplified in eight tumours (3.6 %). *EGFR* gene amplification was more common in tumours of distal oesophagus/gastro-oesophageal junction/cardia than in those of gastric corpus (*p* = 0.013). It was associated with shortened time to cancer recurrence (*p* = 0.026) and cancer specific survival (*p* = 0.033).

**Conclusions:**

*EGFR* gene amplification is relatively common in intestinal adenocarcinomas and associates with decreased survival. It is rarely concurrent with *HER2* gene amplification, suggesting that anti-EGFR therapies might be applicable to some patients not eligible for anti-HER2 treatment. Analogous to HER2 testing, determination of *EGFR* gene amplification status in concert with immunohistochemistry could improve the specificity of patient selection when investigating the possible benefits of anti-EGFR therapies in the treatment of gastric adenocarcinomas.

## Background

EGFR (ERBB1) and HER2 (ERBB2) are members of a tyrosine kinase receptor family frequently activated in cancer either by receptor overexpression or mutations. Metastatic HER2 overexpressing gastric or gastro-oesophageal junction (GOJ) adenocarcinomas can be treated with monoclonal anti-HER2 antibodies in combination with chemotherapy and the only targeted first-line antibody therapy for these tumours is trastuzumab. In contrast, monoclonal anti-EGFR antibodies are currently not indicated for the treatment of gastric cancer, although they are used for patients with metastatic colorectal or head and neck carcinomas.

Gastric adenocarcinomas are traditionally divided into intestinal and diffuse histological subtypes by Laurén classification [1]. Interestingly, it was recently suggested that these tumours can be classified into four distinct molecular subgroups based on their genomic alterations. One of the subgroups, characterised by chromosomal instability (CIN), accounts for about 50 % of gastric cancers and is strongly associated with the intestinal histological subtype and GOJ/cardiac location. Typical alterations in the CIN subtype include *TP53* gene aberrations and activation of the receptor tyrosine kinase–RAS pathway, for example by receptor tyrosine kinase gene amplifications. In contrast, diffuse-type tumours are concentrated in a separate subgroup associating with overall genomic stability as well as distinctive genetic changes affecting cell adhesion and motility [2].

While anti-EGFR antibody treatment is beneficial in colorectal cancer [3, 4], no survival benefit has been observed in phase III clinical trials on gastric and gastro-oesophageal cancer for patients treated with anti-EGFR antibody-chemotherapy combination compared with patients treated with chemotherapy alone [5, 6]. Importantly, however, these studies included no patient selection based on the histological subtype of the tumours, EGFR protein expression or *EGFR* gene copy number (GCN) analysis. As demonstrated in the case of anti-HER2 therapy, an appropriate preselection with an easily applicable biomarker test might increase the potential to identify those patients who could benefit from anti-EGFR therapy.

In this study, we focused on intestinal adenocarcinomas in three locations: the stomach, gastro-oesophageal junction and distal oesophagus. Our aim was to examine the prevalence, clinicopathological associations as well as prognostic role of EGFR and HER2 protein expression and gene amplification in these tumours. First, we analysed EGFR and HER2 alterations by using immunohistochemistry (IHC) to select the tumours with moderate/strong expression of EGFR or HER2 protein. Second, we performed *EGFR* or *HER2* silver *in situ* hybridisation (SISH) in selected cases to quantify GCNs. The validity of this algorithm for *EGFR* gene has previously been demonstrated with colorectal adenocarcinomas [7, 8] and was confirmed in this study by a set of control samples with negative or weak IHC staining.

## Methods

### Patients and clinical tumour material

The study population in this retrospective study consists of 220 patients diagnosed with intestinal adenocarcinoma of the stomach, gastro-oesophageal junction or distal oesophagus at the Turku University Hospital between the years 1993 and 2012. Initially, we used the clinical database of Auria Biobank (see below) to find all patients with the diagnosis of adenocarcinoma of the stomach, gastro-oesophageal junction or distal oesophagus (*n* = 437). The original histopathological information regarding these samples was then obtained to compile a preliminary list of patients, and the respective histological slides were retrieved from the archive. The exclusion criteria for this study were: diffuse or neuroendocrine histological subtype (*n* = 155), metastatic adenocarcinoma from a different organ (*n* = 6), intramucosal carcinoma (Tis) (*n* = 23) and insufficient sample material (*n* = 33). All cases were reanalysed by an expert gastrointestinal pathologist and the intestinal histological subtype of the tumours was confirmed by the presence of well-defined glandular structures in accordance with the Laurén classification [1]. Primarily, tissue samples from primary surgical specimens were included. In order to attain a comprehensive study population, representative biopsies were used in case of 22 patients (10 %): four (1.8 %) patients were not operated due to stage IV disease at the time of diagnosis and 18 (8.2 %) patients had received perioperative chemoradiotherapy resulting in insufficient surgical material for immunohistochemical analysis. The type of surgery was total gastrectomy for 120 (54.5 %) patients, subtotal gastrectomy or tumour resection for 79 (35.9 %) patients and palliative surgery for 17 (7.7 %) patients. The residual tumour classification was determined as R0 (no residual tumour) for 167 (75.9 %) patients, R1 (microscopic residual tumour) for 24 (10.9 %) patients and R2 (macroscopic residual) for 17 (7.7 %) patients. The residual tumour status could not be determined for 12 (5.5 %) patients. The median follow-up time for all patients was 10.5 years. The patient characteristics are presented in Table 1.

**Table 1:** Patient characteristics

	Female, *N* (%)	Male, *N* (%)	All, *N* (%)
Number of patients	79 (35.9)	141 (64.1)	220
Age at diagnosis (years)			
Median	77	72	74
Range	33–93	43–90	33–93
Site of primary tumour			
Distal oesophagus	4 (5.1)	16 (11.3)	20 (9.1)
GOJ/cardia	17 (21.5)	46 (32.6)	63 (28.6)
Corpus	21 (26.6)	44 (31.2)	65 (29.5)
Antrum/pylorus	37 (46.8)	35 (24.8)	72 (32.7)
Tumour differentiation grade			
Grade 1	14 (17.7)	16 (11.3)	30 (13.6)
Grade 2	33 (41.8)	70 (49.6)	103 (46.8)
Grade 3	32 (40.5)	55 (39.0)	87 (39.5)
Stage at diagnosis			
IA	15 (19.0)	18 (12.8)	33 (15.0)
IB	7 (8.9)	19 (13.5)	26 (11.8)
IIA	17 (21.5)	33 (23.4)	50 (22.7)
IIB	14 (17.7)	19 (13.5)	33 (15.0)
IIIA	7 (8.9)	21 (14.9)	28 (12.7)
IIIB	11 (13.9)	19 (13.5)	30 (13.6)
IIIC	1 (1.3)	5 (3.5)	6 (2.7)
IV	7 (8.9)	7 (5.0)	14 (6.4)
Residual tumour classification			
R0 (no residual tumour)	62 (78.5)	105 (74.5)	167 (75.9)
R1 (microscopic residual tumour)	5 (6.3)	19 (13.5)	24 (10.9)
R2 (macroscopic residual tumour)	8 (10.1)	9 (6.4)	17 (7.7)
Rx (unknown)	4 (5.1)	8 (5.7)	12 (5.5)
Perioperative and adjuvant therapy^a^ (*N* = 206)			
Only chemotherapy	7 (9.7)	24 (17.9)	31 (15.0)
Chemoradiotherapy	4 (5.6)	16 (11.9)	20 (9.7)
Only radiation therapy	1 (1.4)	4 (3.0)	5 (2.4)
No adjuvant therapy	58 (80.6)	89 (66.4)	147 (71.4)
Unknown	2 (2.8)	1 (0.7)	3 (1.5)
Tumour recurrence^b^(*N* = 195)			
No recurrence	55 (79.7)	82 (65.1)	137 (70.3)
Single metastasis >6 months	10 (14.5)	26 (20.6)	36 (18.5)
Multiple metastases >6 months	4 (5.8)	18 (14.3)	22 (11.3)
Follow-up status			
Alive and free of disease	22 (27.8)	31 (22.0)	53 (24.1)
Alive with disease	1 (1.3)	1 (0.7)	2 (0.9)
Died of disease	43 (54.4)	74 (52.5)	117 (53.2)
Died of other cause	12 (15.2)	30 (21.3)	42 (19.1)
Unknown cause of death	1 (1.3)	5 (3.5)	6 (2.7)

*GOJ* gastro-oesophageal junction

^a^Excluding stage IV, ^b^Excluding stage IV and recurrence <6 months

Tumour stage was assessed according to the current WHO Classification manual [9]. The study was conducted in accordance with the Declaration of Helsinki and the Finnish legislation for the use of archived tissue specimens and associated clinical information. The clinical data were retrieved, and the histological samples were collected and analysed with the endorsement of the National Authority for Medico-Legal Affairs and The Ethics Committee of the Hospital District of Southwest Finland as well as with the permission of Auria Biobank hosting the specimen archive. All the specimens were from Auria biobank, which has obtained its archived diagnostic sample collection with an opt-out procedure according to the Finnish biobank act [10]. Biobanks authorized and inspected by National Supervisory Authority for Welfare and Health can provide human specimens collected during diagnostic procedures and associated clinical information for research purposes based on the biobank’s scientific board review. Thus, informed consent from surviving patients was not required.

### Procedures

For each tumour, the most representative formalin-fixed paraffin-embedded (FFPE) tissue block was chosen and new sections were cut for both IHC staining and SISH. The methods for EGFR IHC and *EGFR* SISH have been described previously [7], and HER2 IHC was performed similarly with monoclonal HER2 antibody (clone 4B5, Ventana Medical Systems/Roche Diagnostics, Tucson, AZ, USA). *HER2*/Chr17 double-SISH was detected with *HER2* DNA Probe and INFORM Chromosome 17 Probe (Ventana/Roche) and performed with ultraView SISH Detection Kit and ultraView Alkaline Phosphatase (AP) Red ISH Detection Kit (Ventana/Roche).

### Immunohistochemistry and silver *in situ* hybridization

With EGFR, tumour scoring was based on the most intense membranous or membranous + cytoplasmic staining (0, negative; 1+, weak; 2+, moderate; 3+, strong). Strong staining was seen as intense reaction with 5x objective magnification, moderate staining was clearly identified with 5x objective magnification and weak staining was identified only with 10x objective magnification. Specimens were classified as IHC high if showing 2+ or 3+ membranous or membranous + cytoplasmic staining intensity in ≥10 % of tumour cells in surgical specimens or in ≥5 clustered tumour cells in biopsies. These IHC high samples were further analysed with SISH. This algorithm is based on our previous observation that high EGFR IHC staining intensity positively correlates with increased *EGFR* GCN [7]. With HER2 IHC, tumours were scored according to standard criteria [11, 12] and specimens showing 2+ or 3+ membranous staining in ≥10 % of tumour cells or in ≥5 clustered tumour cells in biopsies were classified as IHC high and analysed with SISH. EGFR and HER2 IHC and GCN were scored independently by two observers (EB and JS) without knowledge of the clinical information. Consensus scoring was used in case of differing individual results.

*EGFR* was quantified from the areas of high EGFR IHC intensity as described previously [7, 8]. Forty tumour cells with the highest number of copies were analysed from the EGFR SISH slides and an average value was calculated for each surgical sample. If these forty cells contained numerous overlapping *EGFR* SISH signals (clusters), the tumour was determined to have *EGFR* gene amplification. In biopsies, a group of ≥5 tumours cells with gene clusters was considered as amplification. One *EGFR* cluster was approximated to contain ≥10 gene copies. *HER2* GCN was detected with chromosome 17 (Chr-17) number (number of copies of chromosome per cell) and the *HER2*/Chr-17 ratio was assessed according to standard criteria [13]. If *HER2* gene clusters were detected in ≥10 % of tumour cells in surgical specimens or in a group of ≥5 tumour cells in biopsies, the tumour was determined to contain *HER2* gene amplification. One *HER2* cluster was counted as ≥6 gene copies. To validate our method of including only tumours with high EGFR IHC intensity for *EGFR* SISH, we assessed *EGFR* GCN in fifteen randomly selected tumours in which EGFR IHC was scored as negative/weak. No *EGFR* amplification was found in these tumours (GCN 2.1–3.3).

### Statistical analysis

Statistical analyses were performed with IBM SPSS Statistics for Windows, version 21.0 (IBM Corporation, Armonk, NY). Frequency table data were analysed using the *χ*^2^ test, either with the Pearson *χ*^2^ test or Fisher’s exact test for categorical variables. 2 × 2 tables were used to calculate odds ratios (OR). Kaplan-Meier method and log-rank test as well as Cox’s proportional hazards regression model were used for univariate survival analysis. Multivariate survival analysis was performed by Cox’s proportional hazards regression model. Variables with a *p*-value under 0.2 in univariate analysis were included in the multivariate analyses. Time to recurrence (TTR) was calculated from the time of diagnosis to the time of first recurrence, death of primary cancer or to the last follow-up date. Only recurrences occurring ≥6 months after diagnosis were considered relevant. Earlier detection of a local or distant recurrence was considered likely to present an initially advanced disease. Patients treated with surgery or surgery and adjuvant therapy without disease recurrence ≥6 months after diagnosis were considered curatively treated. Cancer-specific survival (CSS) was calculated from the time of diagnosis to the time of death of primary cancer or the last follow-up date and overall survival (OS) from the time of diagnosis to the time of death of any cause or the last follow-up date. Five patients (2.3 %) who had received trastuzumab treatment for recurrent cancer were excluded from the CSS and OS analyses and additionally 14 patients with stage IV disease (6.4 %) from the TTR analysis. All statistical tests were two-sided and p-values under 0.05 were considered statistically significant.

## Results

### EGFR and HER2 immunohistochemical staining

All 220 tumour samples were analysed with EGFR and HER2 IHC. High membranous or membranous + cytoplasmic EGFR IHC staining intensity (2+/3+) was observed in 72 (32.7 %) of the tumours, while 2+/3+ HER2 IHC staining intensity was present in 31 (14.1 %) tumours. Among these, concurrent high IHC staining intensity of EGFR and HER2 was detected in 14 (6.4 %) tumours. The results from EGFR and HER2 IHC stainings are shown in Table 2.

**Table 2:** Intensity of EGFR and HER2 immunohistochemical stainings in intestinal adenocarcinomas^a^ (*N* = 220)

IHC staining intensity	EGFR, *N* (%)^a^	HER2, *N* (%)^b^	EGFR and HER2, *N* (%)^c^
0/1+	148 (67.3)	189 (85.9)	131 (59.5)
2+/3+	72 (32.7)	31 (14.1)	14 (6.4)

*IHC* immunohistochemistry. 0, negative; 1+ low; 2+ moderate; 3+ strong

^a^According to the most intense membranous or membranous + cytoplasmic staining

^b^According to the most intense membranous staining

^c^Concordant IHC staining intensity. In 75 tumours (34.1 %) IHC staining intensity was discordant

### *EGFR* and *HER2* silver *in situ* hybridisation

**Fig. 1:**
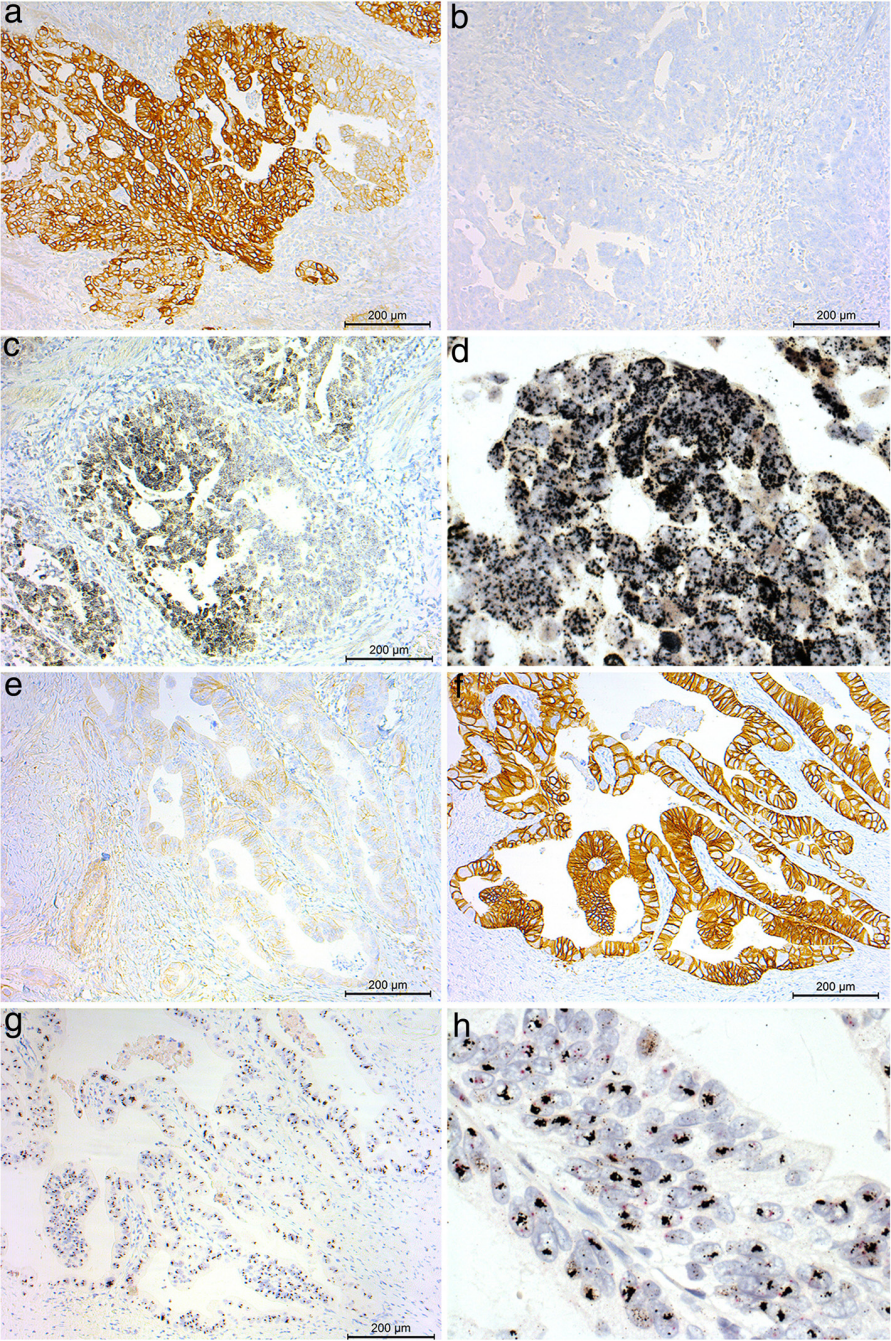
The association between EGFR/HER2 protein expression and *EGFR/HER2* gene amplification in two intestinal-type oesophagogastric adenocarcinomas. Figures **a**–**d** show the same area in a single tumour: **a** Strong (3+) membranous EGFR protein expression (IHC), **b** negative HER2 protein expression and **c**–**d** *EGFR* gene amplification (SISH). Figures **e**–**h** show the same area in another tumour: **e** Negative EGFR protein expression (IHC), (**f**) strong (3+) membranous HER2 protein expression and **g**–**h** *HER2* gene amplification (SISH). Original objective magnification 10x and 60x. IHC, immunohistochemistry; SISH, silver *in situ* hybridisation

**Fig. 2:**
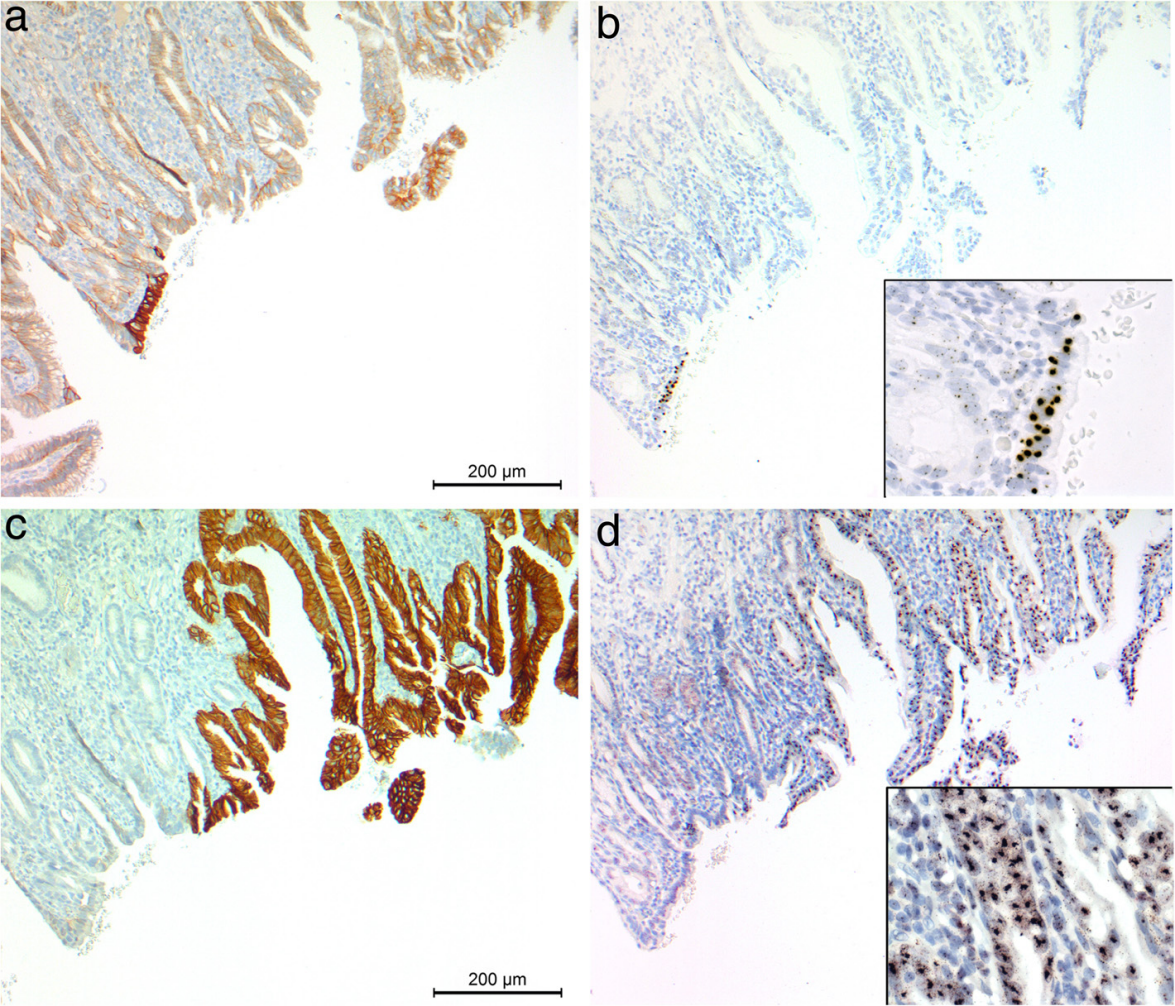
The association between strong EGFR/HER2 protein expression and *EGFR/HER2* gene amplification in a single intestinal-type oesophagogastric adenocarcinoma (original objective magnification 10x). All images are from the same area of the tumour. **a** Strong (3+) EGFR protein expression (IHC). **b** *EGFR* gene amplification (SISH). **c** Strong (3+) HER2 protein expression (IHC). **d** *HER2* gene amplification (SISH). Insets show the gene amplification (original objective magnification 60x). Note that *EGFR* and *HER2* are not amplified in the same cancer cells but in adjacent areas. IHC, immunohistochemistry; SISH, silver *in situ* hybridisation

Gene copy numbers were analysed with *EGFR* or *HER2* SISH in all tumours with high EGFR or HER2 IHC staining intensity. *EGFR* gene amplification was found in 31/72 tumours (14.1 % of the whole study material) and *HER2* gene amplification in 29/31 tumours (13.2 % of the whole study material). Among these, *EGFR* and *HER2* co-amplification was detected in 8/14 tumours (3.6 % of the whole study material). *EGFR* and *HER2* gene amplification status was significantly concordant in antrum (Fisher’s exact test, *p* = 0.004). The results from *EGFR* and *HER2* SISH stainings according to anatomical location are presented in Table 3. There was marked intratumoural heterogeneity of *EGFR* and *HER2* gene amplification, as shown in Figs. 1 and 2.

**Table 3:** *EGFR* and *HER2* silver *in situ* hybridization in intestinal-type adenocarcinomas according to anatomical location

Gene copy number status	Distal oesophagus	GOJ/cardia	Corpus	Antrum/pylorus	Total	*P* value
	*N* = 20 (%)	*N* = 63 (%)	*N* = 65 (%)	*N* = 72 (%)	*N* = 220 (%)	(*χ* ^2^ test)^c^
*EGFR* amplification^a^						
Yes	5 (16.1)	13 (41.9)	2 (6.5)	11 (35.5)	31 (100.0)	0.013^d^
No	15 (7.9)	50 (26.5)	63 (33.3)	61 (32.3)	189 (100.0)	
Total N of amplification (%)	5/20 (25.0)	13/63 (20.6)	2/65 (3.1)	11/72 (15.3)	31/220 (14.1)	
*HER2* amplification^a^						
Yes	5 (17.2)	9 (31.0)	9 (31.0)	6 (20.7)	29 (100.0)	NS
No	15 (7.9)	54 (28.3)	56 (29.3)	66 (34.6)	191 (100.0)	
Total N of amplification (%)	5/20 (25.0)	9/63 (14.3)	9/65 (13.8)	6/72 (8.3)	29/220 (13.2)	
*EGFR* and *HER2* co-amplification^a^						
Yes	2 (25.0)	1 (12.5)	1 (12.5)	4 (50.0)	8 (100.0)	NS
No	18 (8.5)	62 (29.2)	64 (30.2)	68 (32.1)	212 (100.0)	
Total N of co-amplification (%)	2/20 (10.0)	1/63 (1.6)	1/65 (1.5)	4/72 (5.6)	8/220 (3.6)	
*P* value (Fisher’s exact test)^b^	NS	NS	NS	0.004^d^		

*IHC* immunohistochemistry, *GOJ* gastro-oesophageal junction, *GCN* gene copy number, *NS* not significant

^a^Amplification, GCN >10 for *EGFR*; GCN >6 for *HER2*

^b^Concordant *vs.* discordant *EGFR* and *HER2* amplification status

^c^Distal oesophagus, GOJ and cardia *vs.* corpus

^d^Statistically significant

### EGFR and HER2 protein expression and gene amplification in relation to clinicopathological variables

Evaluated by IHC staining intensity, moderate or strong EGFR protein expression was associated with the depth of tumour invasion (pT3–pT4 *versus* pT1–pT2; Fisher’s exact test, *p* = 0.029); OR 2.15, 95 % CI: 1.11–4.17), but did not associate with tumour location (distal oesophagus/GOJ/cardia *versus* gastric corpus/antrum/pylorus; Fisher’s exact test, *p* = 0.054). In contrast, no significant association was found between HER2 protein expression levels and the depth of tumour invasion or tumour location. No significant association was observed between EGFR or HER2 protein expression levels and patient gender, tumour stage or histological differentiation grade.

*EGFR* gene amplification was associated with deep invasion (pT3–pT4 *versus* pT1–pT2; Fisher’s exact test, *p* = 0.020; OR 3.49, 95 % CI: 1.17–10.4) and it was more commonly detected in stage III–IV tumours than in stage I–II tumours (Fisher’s exact test, *p* = 0.024; OR 2.55, 95 % CI: 1.18–5.51). Additionally, *EGFR* gene amplification was more common in tumours of distal oesophagus (5/20 tumours, 25.0 %) and GOJ/cardia (13/63 tumours, 20.6 %) than in those of gastric corpus (2/65 tumours, 3.1 %) (*χ*^2^, *p* = 0.013). This distribution pattern was also seen in male patients (*χ*^2^, *p* = 0.034) but not in female patients. When tumour location was considered as a dichotomous variable, *EGFR* gene amplification was still more common in proximally located tumours (distal oesophagus/GOJ/cardia *versus* gastric corpus/antrum/pylorus; (Fisher’s exact test, *p* = 0.016); OR 2.64, 95 % CI: 1.22–5.73). When analysed separately for males and females, the association between *EGFR* gene amplification and proximal tumours was significant in males (Fisher’s exact test, *p* = 0.011; OR 3.58, 95 % CI: 1.37–9.36) but not in females. In contrast, *HER2* gene amplification status was not significantly associated with the depth of tumour invasion, tumour stage or tumour location. No significant association was found between *EGFR* or *HER2* gene amplification status and patient gender, age at diagnosis or histological differentiation grade of the tumour. The association between EGFR and HER2 protein expression as well as gene amplification and different clinicopathological variables are presented in Table 4.

**Table 4:** Association between the clinicopathological variables and EGFR/HER2 protein expression or gene amplification (*N* = 220)

	EGFR IHC staining intensity, *N* (%)			*EGFR in situ* hybridisation status, *N* (%)			HER2 IHC staining intensity, *N* (%)			*HER2 in situ* hybridisation status, *N* (%)		
	0/1+	2+/3+	*P* value^a^	*EGFR* gene amplification	No *EGFR* gene amplification	*P* value^a^	0/1+	2+/3+	*P* value^a^	*HER2* gene amplification	No *HER2* gene amplification	*P* value^a^
Patient gender												
Female	59 (39.9)	20 (27.8)	NS	8 (25.8)	71 (37.6)	NS	69 (36.5)	10 (32.3)	NS	8 (27.6)	71 (37.2)	NS
Male	89 (60.1)	52 (72.2)		23 (74.2)	118 (62.4)		120 (63.5)	21 (67.7)		21 (72.4)	120 (62.8)	
Site of primary tumour												
Distal oesophagus/GOJ/cardia	49 (33.1)	34 (47.2)	NS	18 (58.1)	65 (34.4)	0.016	69 (36.5)	14 (45.2)	NS	14 (48.3)	69 (36.1)	NS
Corpus/antrum/pylorus	99 (66.9)	38 (52.8)		13 (41.9)	124 (65.6)		120 (63.5)	17 (54.8)		15 (51.7)	122 (63.9)	
Histological differentiation grade												
Grade I	23 (15.5)	7 (9.7)	NS	2 (6.5)	28 (14.8)	NS	28 (14.8)	2 (6.5)	NS	2 (6.9)	28 (14.7)	NS
Grade II	71 (48.0)	32 (44.4)		17 (54.8)	86 (45.5)		83 (43.9)	20 (64.5)		18 (62.1)	85 (44.5)	
Grade III	54 (36.5)	33 (45.8)		12 (38.7)	75 (39.7)		78 (41.3)	9 (29.0)		9 (31.0)	78 (40.8)	
Postoperative T^b^												
pT1–pT2	54 (37.0)	15 (21.4)	0.029	4 (13.31)	65 (34.9)	0.020	63 (34.1)	6 (19.4)	NS	6 (20.7)	63 (33.7)	NS
pT3–pT4	92 (63.0)	55 (78.6)		26 (86.7)	121 (65.1)		122 (65.9)	25 (80.6)		23 (79.3)	124 (66.3)	
Postoperative stage												
I–II	100 (67.6)	42 (58.3)	NS	14 (45.2)	128 (67.7)	0.024	125 (66.1)	17 (54.8)	NS	15 (51.7)	127 (66.5)	NS
III–IV	48 (32.4)	30 (41.7)		17 (54.8)	61 (32.3)		64 (33.9)	14 (45.2)		14 (48.3)	64 (33.5)	

^a^Fisher’s exact test

^b^ *N* = 216, the depth of tumour invasion could not be determined for four patients not receiving surgical treatment

### *EGFR* and *HER2* gene amplification in relation to survival

**Fig. 3:**
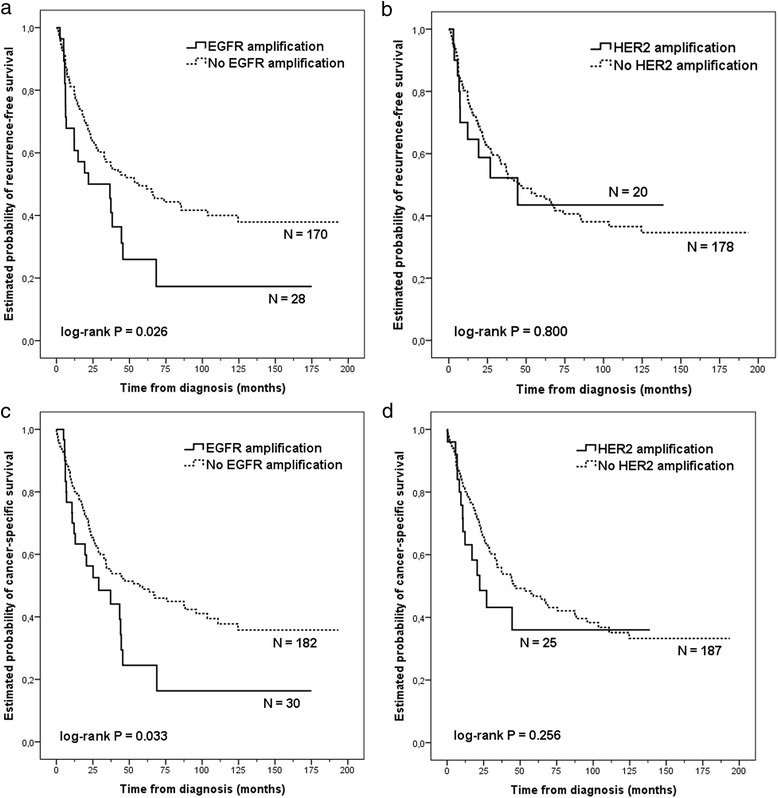
Kaplan-Meier survival curves of intestinal-type oesophagogastric cancer patients with or without *EGFR* or *HER2* amplification. Time to recurrence (**a**–**b**) and cancer-specific survival (**c**–**d**) as based on *EGFR* (**a**, **c**) and *HER2* (**b**, **d**) SISH and IHC analyses. IHC, immunohistochemistry; SISH, silver *in situ* hybridisation

In univariate survival analysis, *EGFR* gene amplification was associated with shortened time to recurrence (TTR, median) (22 *vs.* 57 months, log-rank test, *p* = 0.026; Cox test, *p* = 0.028, HR: 1.73, 95 % CI: 1.06–2.83) and with shortened cancer-specific survival (CSS, median) (29 *vs.* 57 months, log-rank test, *p* = 0.033; Cox test, *p* = 0.035, HR: 1.67, 95 % CI: 1.04–2.69) (Fig. 3). Median TTR and CSS of the patients were both 45 months. *HER2* gene amplification was not significantly associated with TTR, but patients with *HER2* gene amplification had a notably lower median CSS of 22 months than patients without *HER2* amplification (46 months). However, the difference was not statistically significant (log-rank test, *p* = 0.256) (Fig. 3).

In univariate analysis, increasing depth of tumour invasion was associated with decreased TTR and CSS (TTR: log-rank test, *p* < 0.0001; Cox test, *p* < 0.0001, HR 1.46, 95 % CI: 1.19–1.80 and CSS: log-rank test, *p* < 0.0001; Cox test, *p* < 0.0001, HR 1.60, 95 % CI: 1.30–1.96). Similarly, increasing tumour stage was associated with decreased TTR and CSS (TTR: log-rank test, *p* = 0.005; Cox test, *p* = 0.001, HR 1.52, 95 % CI: 1.18–1.96 and CSS: log-rank test, *p* < 0.0001; Cox test *p* < 0.0001, HR 1.94, 95 % CI: 1.53–2.45). In addition, increasing patient age at the time of diagnosis was associated with shorter CSS (Cox test, *p* = 0.048, HR 1.02, 95 % CI: 1.00 − 1.04), but not with TTR (Cox test, *p* = 0.341). No significant association was found between patient gender (log-rank test, TTR: *p* = 0.372; CSS: *p* = 0.818) or tumour location (log-rank test, TTR: *p* = 0.057; CSS: *p* = 0.262). In Kaplan-Meier analysis, histological differentiation grade was not associated with survival (grade I *versus* II *versus* III; log-rank test, TTR: *p* = 0.118; CSS: *p* = 0.053). However, when analysed separately grade II tumours were associated with shorter TTR in comparison to grade I tumours (univariate Cox test, *p* = 0.043, HR 1.95, 95 % CI: 1.02–3.74). Additionally, grade II and III tumours were associated with shorter CSS in comparison to grade I tumours (univariate Cox test, grade II: *p* = 0.020, HR 2.22, 95 % CI: 1.13–4.36; grade III: *p* = 0.029, HR 2.15, 95 % CI: 1.08–4.27). No significant association was observed between *EGFR* or *HER2* gene amplification status and overall survival (OS). EGFR or HER2 protein expression, evaluated by IHC staining intensity, was not significantly associated with TTR, CSS or OS.

In the multivariate model for TTR, *EGFR* gene amplification was analysed together with tumour stage, histological differentiation grade and tumour location. In the multivariate analysis for CSS, *EGFR* gene amplification was analysed together with tumour stage, histological differentiation grade and patient age at the time of diagnosis. Tumour stage remained as a single predictive factor for TTR (Cox test, stage III: *p* = 0.014, HR 2.05, 95 % CI: 1.16–3.63) as well as for CSS (Cox test, stage III: *p* = 0.023, HR 1.99, 95 % CI: 1.10–3.61; stage IV: *p* < 0.0001, HR 11.4, 95 % CI: 5.34–24.4). The results from univariate and multivariate survival analyses are presented in Table 5.

**Table 5:** Time to recurrence (TTR)^a^ and cancer-specific survival (CSS)^b^ of patients with intestinal-type adenocarcinomas

	Univariate survival analysis for TTR						Multivariate survival analysis for TTR			Univariate survival analysis for CSS						Multivariate survival analysis for CSS		
	Number of patients	TTR, median (months)	*P* value, log-rank test^c^	*P* value, Cox test^d^	HR	95 % CI	*P* value, Cox test^b^	HR	95 % CI	Number of patients	CSS, median (months)	*P* value, log-rank test^a^	*P* value, Cox test^d^	HR	95 % CI	*P* value, Cox test^b^	HR	95 % CI
Age (continuous variable)	198			NS						212			0.048	1.02	1.00–1.04	NS		
Patient gender																		
Female (reference)	71	56.6	NS	NS						78	45.6	NS	NS					
Male	127	38.2								134	44.6							
Site of primary tumour																		
Distal oesophagus/GOJ/cardia (reference)	75	28.5	NS	NS						77	34.3	NS						
Corpus/antrum/pylorus	123	53.6					NS			135	47.1		NS					
Histological differentiation grade																		
Grade I (reference)	30	NA	NS							30	NA	NS						
Grade II	92	33.8		0.043	1.95	1.02–3.74	NS			98	33.8		0.020	2.22	1.13–4.36	NS		
Grade III	76	53.2		NS			NS			84	44.6		0.029	2.15	1.08–4.27	NS		
Postoperative T																		
pT1 (reference)	37	67.3	<0.0001							37	NA	<0.0001						
pT2	31	NA		NS						31	NA		NS					
pT3	75	56.6		NS						82	57.3		NS					
pT4	55	20.5		0.002	2.59	1.44–4.67				59	25.7		0.001	2.94	1.58–5.47			
Postoperative stage																		
I (reference)	58	NA	0.005							58	NA	<0.0001						
II	80	38.2		NS			NS			80	57.3		NS			NS		
III	60	22.6		0.001	2.33	1.38–3.92	0.014	2.05	1.16–3.63	60	29.0		0.002	2.36	1.37–4.08	0.023	1.99	1.10–3.61
IV	NA									14	6.90		<0.0001	14.2	6.86–29.3	<0.0001	11.4	5.34–24.4
*EGFR* amplification																		
Yes	28	21.8	0.026	0.028	1.73	1.06–2.83	NS			30	29.0	0.033	0.035	1.67	1.04–2.69	NS		
No (reference)	170	56.6								182	57.3							
*HER2* amplification																		
Yes	20	44.6	NS	NS						25	22.3	NS	NS					
No (reference)	178	45.6								187	45.6							

*NS* not significant, *NA* not applicable

^a^Excluding trastuzumab-treated and stage IV patients

^b^Excluding trastuzumab-treated patients

^c^Kaplan-Meier method

^d^Cox’s proportional hazards regression model

## Discussion

This study shows that *EGFR* gene amplification is not uncommon in intestinal adenocarcinoma of the stomach, gastro-oesophageal junction and distal oesophagus. In addition, we demonstrate that *EGFR* amplification is most prevalent in proximally located tumours and significantly associated with decreased survival, as defined by TTR and CSS.

In previous studies, *EGFR* gene amplification has been reported to be present in only 2.3–4.9 % of gastric cancers including all histological subtypes [14–16], whereas the reported numbers for *HER2* gene amplification vary between 7 and 17 % [17, 18]. The prevalence of *EGFR* and *HER2* co-amplification has been reported as low (<0.5 %) [15, 16], albeit studies analysing concurrent *EGFR* and *HER2* GCN changes are few and none have been carried out after the novel molecular subtypes of gastric cancer were published [2]. In contrast, we found *EGFR* gene amplification in 14.4 % and receptor co-amplification in 3.6 % of intestinal adenocarcinomas.

HER2 has been found to be overexpressed, as determined by both IHC and GCN analyses, in 7–25 % of gastric adenocarcinomas [11, 12, 17, 19] including all histological subtypes, which is comparable with our finding that high HER2 protein expression was found in 14.1 % and *HER2* gene amplification in 13.2 % of intestinal adenocarcinomas.

Recent molecular classification studies have linked approximately 36–50 % of gastric adenocarcinomas with characteristics such as intestinal-type histology, chromosomal abnormalities, changes in the receptor tyrosine kinase–RAS signaling pathway, as well as *TP53* gene and somatic copy-number aberrations. These characteristics have been associated with a distinct molecular subgroup: tumours in the CIN subgroup are characterised by chromosomal instability, while the MSS/TP53^−^ subgroup typically contains microsatellite stable tumours with inactive TP53 [2, 20]. Both of these studies could further show that histologically diffuse-type tumours are concentrated in a separate subgroup with molecular characteristics different from those defining CIN or MSS/TP53^−^. However, the predominant anatomical location of tumours belonging to either CIN or MSS/TP53^−^ subgroup was found to differ: CIN tumours were mostly located in GOJ/cardia, whereas MSS/TP53^−^ tumours were predominantly situated in gastric antrum [2, 20]. It has been previously demonstrated that *HER2* gene amplification is strongly associated with the intestinal histological subtype, as compared to the diffuse subtype, as well as with the gastro-oesophageal location of tumours [17, 19]. In our material, *EGFR* gene amplification was most common in the tumours of distal oesophagus and GOJ/cardia, as observed in the CIN subgroup, but infrequent in the tumours of gastric corpus. In antral/pyloric tumours, the observed prevalence of *EGFR* gene amplification was intermediate to that in other locations.

*EGFR* gene amplification was found to be significantly associated with decreased TTR and CSS, which is consistent with earlier findings of association between *EGFR* gene amplification and survival [14, 15]. Results from these studies are, however, based on notably smaller sample size and/or histologically more heterogeneous tumour material than included in this present study. There are contradictory reports regarding the relevance of *HER2* gene amplification as a negative prognostic factor in gastric cancer [15, 17]. In this study, the non-significant association may partly be related to including only intestinal adenocarcinomas in the study material.

HER2 overexpression is known to predict treatment benefit from anti-HER2 antibody therapy. The survival of patients is significantly improved in metastatic gastric and gastro-oesophageal cancer by the addition of trastuzumab to a cisplatin-fluoropyrimidine-containing chemotherapy regimen [12], whereas no survival benefit has been demonstrated in phase III clinical trials with anti-EGFR antibody treatment in comparison to other chemotherapeutic regimens [5, 6]. While the EGFR status was not used for patient selection in these earlier studies, an ongoing phase III clinical trial has been reported to select patients based on EGFR overexpression, although defined only by IHC [21]. Overexpression of EGFR protein has been reported in 24–27 % of all gastric adenocarcinomas [14, 16] and in 31 % [14] of intestinal gastric adenocarcinomas. In our study, we found that 32.7 % of the intestinal adenocarcinomas had high EGFR IHC staining intensity, but only 31/72 (43.1 %) of these demonstrated *EGFR* gene amplification. This suggests that determining EGFR overexpression of tumours only by IHC, without knowledge of the *EGFR* GCN, may be an inadequate method for selecting patients for anti-EGFR therapy. Indeed, a recent preclinical study with patient derived xenografts indicated that strongest response to anti-EGFR therapy was achieved in tumours with *EGFR* gene amplification [22]. The relatively low prevalence of co-amplification of *EGFR* and *HER2* genes (3.6 % in this study) demonstrates the presence of two distinct subgroups of patients with either *EGFR* or *HER2* gene amplification, which implies that anti-EGFR therapies might be applicable to some patients not eligible for anti-HER2 treatment. Those patients having receptor co-amplification might even benefit from a dual-acting antibody treatment.

## Conclusions

In this study, we have shown that *EGFR* gene amplification is relatively common in intestinal adenocarcinomas of the stomach, gastro-oesophageal junction and distal oesophagus and associates with decreased survival. We have also demonstrated that *EGFR* GCN can be easily analysed by silver *in situ* hybridisation in diagnostic tumour material and thus could be applied as a routine histopathological diagnostic method. Based on our results, we suggest that determining *EGFR* gene amplification status in concert with IHC could be used in future clinical trials to identify patients with inverse prognosis and to improve the specificity of patient selection when investigating the possible benefits of anti-EGFR therapies in the treatment of intestinal-type gastro-oesophageal adenocarcinomas.

## Abbreviations

Chr-17, chromosome 17; CI, confidence interval; CIN, chromosomal instability; CSS, cancer-specific survival; EGFR (ERBB1), epidermal growth factor receptor (erb-b2 receptor tyrosine kinase 1); FFPE, formalin-fixed paraffin-embedded; GCN, gene copy number; GOJ, gastro-oesophageal junction; HER2 (ERBB2), human epidermal growth factor receptor 2 (erb-b2 receptor tyrosine kinase 2); HR, hazard ratio; IHC, immunohistochemistry; MSS, microsatellite stable; NA, not applicable; NS, not significant; OR, odds ratio; OS, overall survival; pT, pathologic T, describes tumour size and depth of invasion; RAS, rat sarcoma viral oncogene homolog; SISH, silver *in situ* hybridization; TP53, tumour protein p53; TTR, time to recurrence.
